# Genetic Polymorphisms Associated With the Pharmacokinetics, Pharmacodynamics and Adverse Effects of Olanzapine, Aripiprazole and Risperidone

**DOI:** 10.3389/fphar.2021.711940

**Published:** 2021-07-14

**Authors:** Paula Soria-Chacartegui, Gonzalo Villapalos-García, Pablo Zubiaur, Francisco Abad-Santos, Dora Koller

**Affiliations:** ^1^ Clinical Pharmacology Department, School of Medicine, Hospital Universitario de La Princesa, Instituto Teófilo Hernando, Universidad Autónoma de Madrid, Instituto de Investigación Sanitaria La Princesa (IP), Madrid, Spain; ^2^ UICEC Hospital Universitario de La Princesa, Platform SCReN (Spanish Clinical Research Network), Instituto de Investigación Sanitaria La Princesa (IP), Madrid, Spain; ^3^ Centro de Investigación Biomédica en Red de Enfermedades Hepáticas y Digestivas (CIBERehd), Instituto de Salud Carlos III, Madrid, Spain; ^4^ Department of Psychiatry, Yale School of Medicine and VA CT Healthcare Center, West Haven, CT, United States

**Keywords:** antipsychotics, pharmacogenetics, metabolism, genetic polymorphism, pharmacokinetics

## Abstract

Olanzapine, aripiprazole and risperidone are atypical antipsychotics or neuroleptics widely used for schizophrenia treatment. They induce various adverse drug reactions depending on their mechanisms of action: metabolic effects, such as weight gain and alterations of glucose and lipid metabolism; hyperprolactinemia and extrapyramidal effects, such as tremor, akathisia, dystonia, anxiety and distress. In this review, we listed polymorphisms associated with individual response variability to olanzapine, aripiprazole and risperidone. Olanzapine is mainly metabolized by cytochrome P450 enzymes, CYP1A2 and CYP2D6, whereas aripiprazole and risperidone metabolism is mainly mediated by CYP2D6 and CYP3A4. Polymorphisms in these genes and other enzymes and transporters, such as enzymes from the uridine 5'-diphospho-glucuronosyltransferase (UGT) family and ATP-binding cassette sub-family B member 1 (ABCB1), are associated to differences in pharmacokinetics. The three antipsychotics act on dopamine and serotonin receptors, among others, and several studies found associations between polymorphisms in these genes and variations in the incidence of adverse effects and in the response to the drug. Since olanzapine is metabolized by CYP1A2, a lower starting dose should be considered in patients treated with fluvoxamine or other CYP1A2 inhibitors. Regarding aripiprazole, a reduced dose should be administered in CYP2D6 poor metabolizers (PMs). Additionally, a reduction to a quarter of the normal dose is recommended if the patient is treated with concomitant CYP3A4 inhibitors. Risperidone dosage should be reduced for CYP2D6 PMs and titrated for CYPD6 ultrarapid metabolizers (UMs). Moreover, risperidone dose should be evaluated when a CYP2D6, CYP3A4 or ABCB1 inhibitor is administered concomitantly.

## Introduction

Schizophrenia is a debilitating mental illness characterized by a distortion of thinking, perceptions, emotions and behavior. It is a complex syndrome that begins in adolescence and early adulthood, affecting daily functioning and educational and work performance. One percent of the world's population suffers from schizophrenia, establishing an urgent problem for healthcare systems worldwide (Schultz et al., 2007). It is a polygenic disorder influenced by environmental factors. The symptoms of this illness are classified into positive, negative and cognitive. Positive symptoms, also called psychotic symptoms, include hallucinations and delusions. The main negative symptoms include flattened affect, anhedonia, apathy, social withdrawal and loss of will. Cognitive impairment includes attention, memory and language deficit and disorientation (Mueser and McGurk, 2004).

Schizophrenia is developed by alterations in different neurotransmitters (Laruelle, 2014). The main hypothesis is the dopaminergic hypothesis, which proposes a chemical imbalance of dopamine in the brain (Brisch et al., 2014). Nevertheless, alternative hypotheses were developed that complement the dopaminergic hypothesis and that are related to other neurotransmitters, such as serotonin or glutamate (Laruelle, 2014; Stahl, 2018). Consequently, the main targets of antipsychotic drugs which are used for schizophrenia treatment are dopamine and serotonin receptors. These antipsychotics, also referred as neuroleptics, are classified into typical and atypical, and differ in their mechanism of action, therefore the side effects they induce (Meltzer, 2013). Typical antipsychotics, e.g., haloperidol, which act mainly as dopamine receptor (DRD) antagonists, have little or no effect on negative and cognitive symptoms and frequently cause extrapyramidal adverse effects (Maric et al., 2016). Atypical antipsychotics, also called second generation antipsychotics (SGAs), act as antagonists to DRD, serotonin (5-HTR), histamine, α-adrenergic and muscarinic receptors. The most common SGAs are olanzapine, quetiapine, clozapine, risperidone and paliperidone. These antipsychotics are associated with metabolic adverse effects and weight gain. In addition, some authors propose that aripiprazole, a SGA with unique mechanism of action, is a third generation antipsychotic as it is a partial agonist of DRD2 (Maric et al., 2016). Moreover, antipsychotic drugs are also used for the treatment of acute manic or mixed episodes associated with bipolar disorder, for the major depression disorder and for autistic disorder (FDA, 1993, 1996, 2014).

This review provides a comprehensive summary of pharmacogenetic biomarkers associated with the metabolic effects of three representative atypical antipsychotics: risperidone, olanzapine and aripiprazole. Aripiprazole was selected since it has a unique mechanism of action. Risperidone and olanzapine were selected for being representative of two of the main types of atypical antipsychotics.

## Mechanism of Action and Metabolism of Olanzapine, Aripiprazole and Risperidone

Olanzapine and risperidone are atypical antipsychotics that act as antagonists at different neurotransmitters receptors, such as DRD, 5-HTR, histamine, α-adrenergic and muscarinic receptors. Aripiprazole acts as a partial agonist of DRD2 and 5-HTR (Maric et al., 2016). The complete mechanism of action of olanzapine, risperidone and aripiprazole is shown in Table 1. However, the mechanism of action of antipsychotics is not completely understood. Therefore, the barrier between side effects and adverse events is questionable as their mechanism of action is poorly described.

**TABLE 1:** Mechanism of action of olanzapine, risperidone and aripiprazole.

Drug	Year of approval	Usual effective dose	Mechanism of action
Risperidone	1993	4 mg once daily	Antagonist at DRD2, 5-HT_2A_, 5-HT_2C_, α_1_ and α_2_ adrenergic and H_1_ histaminergic receptors (Germann et al., 2012)
Olanzapine	1996	10 mg once daily	Antagonist of DRD2, DRD3, DRD4, serotonin 5-HT_2A_, 5-HT_2B_, 5-HT_2C_, 5-HT_3_, 5-HT_6_, histamine H1, α1-adrenergic and M1-M5 muscarinic receptors (Bhana et al., 2001)
Aripiprazole	2002	20 mg once daily	Partial agonist at dopamine D_2_ and D_3_ receptors and serotonin 5-HT_1A_, 5-HT_2A_, 5-HT_2C_ and 5-HT_7_ receptors; inverse agonist at 5-HT_2B_ receptors (Shapiro et al., 2003)
			Antagonist activity with limited affinity for α1A adrenergic, H1 histaminic and 5-HT_6_ receptors and very low affinity for α2 adrenergic receptors and M1 muscarinic cholinergic receptors (Kinghorn and McEvoy, 2005)

a Aripiprazole shows special atypicality compared to other atypical antipsychotics; it acts as partial agonists at dopamine D2 and D3 receptors and 5-HT_2A_ receptor antagonists (Maric et al., 2016).

### Metabolism

#### Olanzapine

Olanzapine has a 60% of oral bioavailability and displays linear pharmacokinetics over the clinical dosing range. Its bioavailability is not influenced by food intake (Callaghan et al., 1999). It is extensively eliminated by first pass metabolism, with approximately 40% of the dose metabolized before reaching the systemic circulation. Its maximum plasma concentration (C_max_) is reached in 5 h (T_max_) following an oral dose (Tamminga and Kane, 1997). When administered once daily, it requires approximately 1 week to reach steady-state concentrations. Olanzapine is extensively distributed throughout the body, with a volume of distribution (Vd) of approximately 1533 L or 21 L/kg 93% of the drug binds to plasma proteins, primarily to albumin and α1-acid glycoprotein (Callaghan et al., 1999). It is mainly excreted in urine (57%) and feces (30%) (Callaghan et al., 1999). Olanzapine is highly metabolized, since 7% of the drug is excreted as unchanged drug in urine (FDA, 1996). Its half-life (T_1/2_) ranges from 21 to 54 h and its clearance (Cl), from 12 to 47 L/h. Sex, smoking and age affect olanzapine plasma concentrations, Cl and T_1/2_ (Callaghan et al., 1999).

**FIGURE 1:**
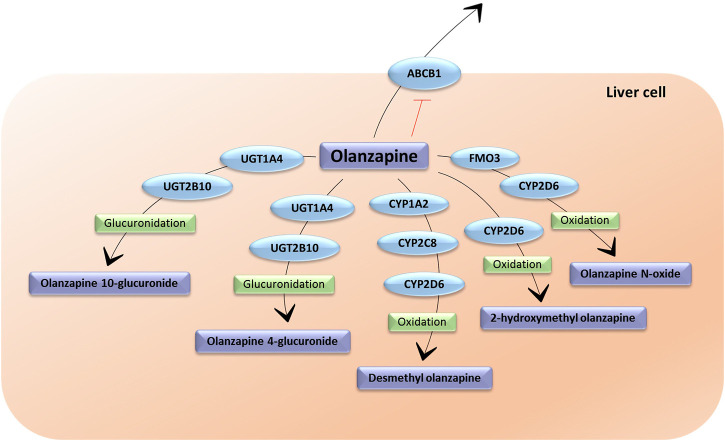
The metabolic pathways of olanzapine. UGT1A4: Uridine 5′-diphospho-glucuronosyltransferase Family 1 Subfamily A Member 4; UGT2B10: Uridine 5′ diphospho-glucuronosyltransferase Family 2 Subfamily B Member 10; FMO3: flavin monooxigenase 3; ABCB1: ATP-binding cassette sub-family B member 1; CYP1A2: cytochrome P450 Family 1 Subfamily A Member 2; CYP2C8: cytochrome P450 Family 2 Subfamily C Member 8; CYP2D6: cytochrome P450 Family 2 Subfamily D Member 6 (Figure inspired by PharmGKB olanzapine pharmacokinetic pathway).

The metabolism of olanzapine is extensive and complex (Figure 1). It generates different metabolites with a lower pharmacological activity compared to the parent compound, therefore, they do not significantly contribute to the clinical effects of olanzapine (Söderberg and Dahl, 2013). The main reaction olanzapine undergoes is N-glucuronidation, which results in the 10 N-glucuronide and, to a lesser extent, 4 N-glucuronide metabolites. The 10 N-glucuronide metabolite is the main metabolite circulating in plasma, present at steady state at 44% of olanzapine concentration (FDA, 1996). These reactions are mainly catalyzed by uridine diphosphate glucuronosyl transferase UGT1A4 and UGT2B10 (Söderberg and Dahl, 2013). In addition, olanzapine undergoes oxidation reactions catalyzed by different isoforms of the cytochrome P450 enzymes (CYP). The 1A2 isoform (CYP1A2) produces the N-desmethylolanzapine metabolite (DMO), which is present at 31% of the concentration of olanzapine at steady state. DMO is also produced by CYP2D6 and CYP2C8, although to a lesser extent. CYP2D6 and secondarily, CYP3A4 and CYP2C9, produce the 2-hydroximethylolanzapine metabolite (Söderberg and Dahl, 2013; Korprasertthaworn et al., 2015). However, CYP2D6 metabolism seems to occur marginally *in vivo* (FDA, 1996). In addition, olanzapine undergoes N-oxidation reactions, catalyzed by the flavin monooxygenase 3 (FMO3) enzyme, which produces N-oxide olanzapine, also generated by CYP2D6 (Figure 1) (Söderberg and Dahl, 2013; Okubo et al., 2016).

The prevalence of smokers is higher in schizophrenic patients than in general population (52.9% and 40.1%, respectively, *p* = 0.031) (Ohi et al., 2019). Tobacco induces CYP1A2 activity, what leads to reduced olanzapine plasma concentration. Thus, smoking affects olanzapine Cl, however, dosage modifications are not routinely recommended (FDA, 1996; Tsuda et al., 2014).

Olanzapine is transported by and acts as inhibitor of the ATP-binding cassette sub-family B member 1 transporter (ABCB1). This transporter, also called P-glycoprotein (P–gp), is an efflux pump involved in the excretion of several antipsychotics in the blood–brain barrier (BBB), limiting their bioavailability in the brain (Wang et al., 2006). Moreover, it is located in other organs, such as the gastrointestinal tract and the liver, influencing olanzapine location (Wang et al., 2006).

#### Aripiprazole

Aripiprazole has an absolute oral bioavailability of 87%. Depending on the dose, the C_max_ is reached between 2.8 and 6.8 h after drug intake, with a median T_max_ of 3–4 h. It presents linear pharmacokinetics. Its Vd is of 404 L or 4.9 L/kg and it has high affinity for plasma proteins with 99% binding. Cl is 0.7 L/min/kg and is eliminated in feces (60%) and urine (27%) (Shapiro et al., 2003).

**FIGURE 2:**
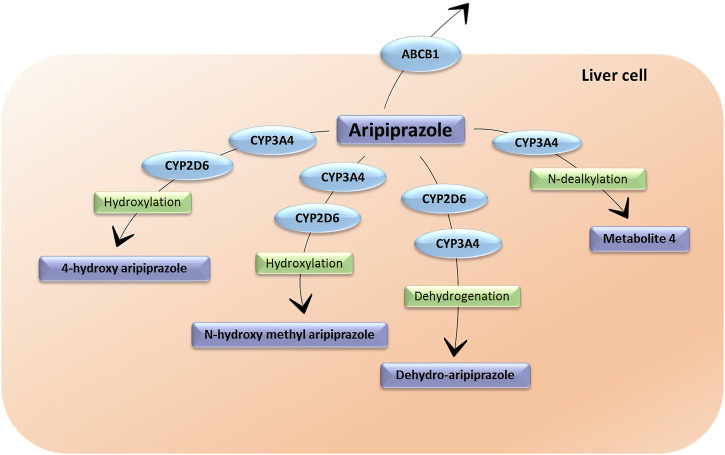
The metabolic pathways of aripiprazole. CYP3A4: cytochrome P450 Family 3 Subfamily A Member 4; CYP2D6: cytochrome P450 Family 2 Subfamily D Member 6. ABCB1: ATP-binding cassette sub-family B member 1. Metabolite 4 does not have an assigned name.

Aripiprazole is mainly transformed in the liver through dehydrogenation, hydroxylation and N-dealkylation by CYP2D6 and CYP3A4 (Figure 2) (Kinghorn and McEvoy, 2005). Its main and only active metabolite is dehydro-aripiprazole, which amounts to 40% of aripiprazole concentration at steady state, reached after 14 days of treatment (Kinghorn and McEvoy, 2005). The T_1/2_ varies between 58 and 78 h and can reach 140 h in CYP2D6 poor metabolizers (PMs) (Kinghorn and McEvoy, 2005). Both aripiprazole and dehydro-aripiprazole are possible substrates of P–gp (Belmonte et al., 2018).

#### Risperidone

Risperidone has an absolute oral bioavailability of 70%. Plasma concentrations of risperidone, its major metabolite and both together are dose proportional over the dosing range (0.5–8 mg twice daily) (FDA, 1993). Following administration, risperidone C_max_ is reached after about 1 h 9-hydroxyrisperidone T_max_ depends on CYP2D6 phenotype: 3 h in normal metabolizers (NM), and 13 h in PM (FDA, 1993). The Vd is 1–2 L/kg. In plasma, 90% of risperidone and 77.4% of 9-hydroxirisperidone is bound to albumin and α1-acid glycoprotein. Risperidone and its metabolites are eliminated via the urine and, to a much lesser extent, via the feces (Germann et al., 2012). The pharmacokinetics of risperidone and 9-hydroxyrisperidone combined, after single and multiple doses, are similar in NM and PM, with an overall mean T_1/2_ of about 20 h (FDA, 1993).

**FIGURE 3:**
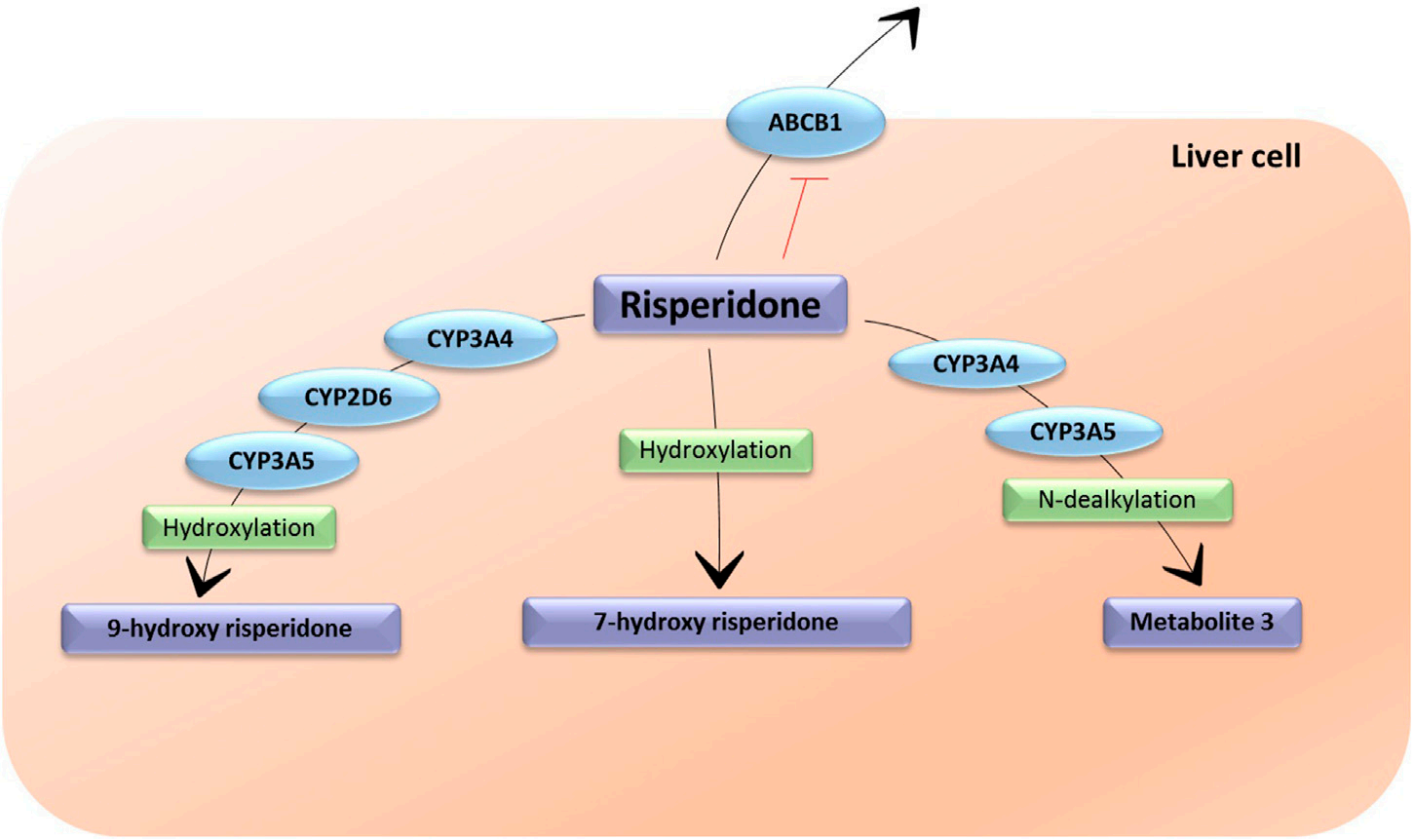
The metabolic pathways of risperidone. CYP3A4: Cytochrome P450 Family 3 Subfamily A Member 4; CYP3A5: Cytochrome P450 Family 3 Subfamily A Member 5; CYP2D6: Cytochrome P450 Family 2 Subfamily D Member 6; ABCB1: ATP-binding cassette sub-family B member 1. Metabolite 3 does not have an assigned name. The enzymes responsible for the formulation of 7-hydroxy risperidone are unknown.

Risperidone suffers a 9-hydroxylation in the liver catalyzed by CYP2D6, CYP3A4 and CYP3A5 (Fang et al., 1999). This hydroxylation produces 9-hydroxyrisperidone, which is an active metabolite that was commercialized as paliperidone (Corena-McLeod, 2015). The sum of risperidone and 9-hydroxyrisperidone represents the active moiety of this antipsychotic. A minor metabolic pathway is through N-dealkylation through CYP3A4 and CYP3A5 (Germann et al., 2012; Ganoci et al., 2021) and 7-hydroxylation (Berecz et al., 2004), both resulting in inactive metabolites. *In vitro* studies showed that risperidone is a substrate and an inhibitor of P-gp (Ganoci et al., 2021) (Figure 3).

## Metabolic Effects of Risperidone, Olanzapine and Aripiprazole

Olanzapine causes metabolic adverse effects to the greatest extent among antipsychotics *e.g.*, weight gain and alterations in glucose and lipid metabolism (Rummel-Kluge et al., 2010; Roerig et al., 2011; Barton et al., 2020). The metabolic effects of risperidone are also notable, but less frequent compared to olanzapine (FDA, 1993; Rummel-Kluge et al., 2010). On the contrary, aripiprazole barely causes these effects (Bak et al., 2014; Barton et al., 2020). The mechanisms by which olanzapine and risperidone cause increased intake, weight gain and fat deposition metabolism are not completely known. It seems to involve different peptide, neurotransmitter and receptor systems in the appetite and reward systems in the brain (Roerig et al., 2011; Aringhieri et al., 2018). A clinical trial reported that olanzapine- and risperidone-induced long term weight gain was not correlated with appetite alterations in chronically treated patients. Nonetheless, the authors did not exclude that weight gain in first time treated patients could be produced by increased appetite (Smith et al., 2012). It is hypothesized that additional drug-mediated mechanisms are partially responsible for antipsychotic-induced weight gain (Himmerich et al., 2015). Olanzapine-induced weight gain was observed even after only 5 days acute treatment in healthy volunteers (Koller et al., 2021). Other studies observed a 0.9 kg per month weight gain in schizophrenic patients treated with olanzapine (Lieberman et al., 2005) or a weight gain of 13.9 kg in patients after 1 year of olanzapine treatment (Kahn et al., 2008).

Risperidone-induced weight gain or metabolic impairments are less frequent and severe compared to olanzapine, but greater compared to aripiprazole (Komossa et al., 2009, 2011; Aringhieri et al., 2018). Clinical trials demonstrated that the same phenomenon occurs in different populations. In 107 adult schizophrenic stable outpatients, olanzapine induced greater weight gain than risperidone, however, risperidone was associated with more hospitalizations (Noordsy et al., 2017). In 144 antipsychotic naïve young patients, adverse changes in adiposity and insulin sensitivity were observed during 12 weeks of antipsychotic treatment with olanzapine, risperidone or aripiprazole, and olanzapine-related fat increase was the most significant (Nicol et al., 2018). An overweight (body mass index (BMI) superior to 26 kg/m^2^) adult cohort of patients presented reduced prevalence of overweight in the risperidone group compared to those receiving olanzapine (Meyer et al., 2005). In a Chinese cohort of first episode schizophrenia patients, aripiprazole was the less weight-gain inducer, as opposed to olanzapine, which was the greater weight gain inducer (Cheng et al., 2019). In contrast to this, other studies reported no significant differences in metabolic syndrome development induced by olanzapine or risperidone. In a clinical trial of 46 overweight patients, olanzapine and risperidone showed no difference in the prevalence of diabetes or prediabetic glucose or glycohemoglobin levels after 5 months of treatment. Nonetheless, olanzapine induced higher insulin resistance and consequently, increased insulin levels (Smith et al., 2009). In the same population, no difference was found in weight-gain after 5 months, although at 2 months of treatment olanzapine induced greater increase in triglycerides, and very low density (VLDL) cholesterol and triglycerides (Smith et al., 2010). Despite these facts, risperidone was found to induce metabolic side effects such as lipid metabolism imbalance, cholesterol and VLDL level increase, hyperglycemia and insulin resistance in other studies (Robinson et al., 2015; Nicol et al., 2018).

One of the receptors that might be involved in the development of metabolic effects is the 5-HT_2A_ serotonin receptor. The blockade of this receptor by olanzapine causes an increase in food intake and weight, by altering the secretion of the neuropeptide PY (Ruaño et al., 2007). In addition, this receptor is involved in the regulation of glucose intake by the muscle, so that its inhibition favors the development of hyperglycemia and insulin resistance (Roerig et al., 2011). On the other hand, blockage of the 5-HT_2C_ receptor causes alterations in satiety and food intake by altering the regulation of leptin (Reynolds et al., 2006). Olanzapine and risperidone are antagonists to 5-HTR and aripiprazole is a partial agonist (Roerig et al., 2011; Aringhieri et al., 2018); therefore the signaling cascade that leads to weight gain via 5-HT receptors may be less stimulated by aripiprazole. Thus, the different affinity profile of aripiprazole could explain the reduced number of metabolic adverse events compared to other antipsychotics (Shapiro et al., 2003; Kane et al., 2009; Rummel-Kluge et al., 2010).

The role of DRD1, DRD2 and DRD3 in weight gain is not clearly known. Nonetheless, presumably olanzapine’s DRD2 antagonism alters feeding behavior since this receptor is involved in the reward system (Rivera-Iñiguez et al., 2019). The blockage of H1 histamine receptor and α1 and α2 adrenergic receptors by olanzapine may alter feeding and glucose control and regulation of body energy balance, playing a role in weight gain and metabolism (Roerig et al., 2011). Aripiprazole and risperidone present moderate affinity for H1 histamine receptors, which may explain their moderate effect on weight gain (Shapiro et al., 2003; Aringhieri et al., 2018).

Olanzapine is also known to increase the risk of developing type II diabetes (Lambert et al., 2006; Koller et al., 2021). Its M3 receptor antagonism causes an alteration in glucose control and insulin response, and the blockade of the H1 receptor favors the development of insulin resistance. Inhibition of 5-HT receptors, in particular 5-HT_1A_, leads to alterations in glucose metabolism favoring hyperglycemia (Jeon and Kim, 2017). Moreover, atypical antipsychotics can also provoke dyslipemia or hyperlipidemia, however, the mechanisms by which this occurs are not clearly known (Jeon and Kim, 2017). It is proposed that olanzapine alters hepatic lipid metabolism by interfering with lipogenesis, lipoprotein internalization and cholesterol clearance, favoring the accumulation of lipids and the development of hyperlipidemia (Chen et al., 2018).

## Adverse Drug Reactions to Olanzapine, Aripiprazole and Risperidone

The most common (≥5% and at least twice than placebo) adverse events to olanzapine in schizophrenic patients are constipation, weight gain, dizziness, personality disorder, akathisia, postural hypotension, sedation, headache, increased appetite, fatigue, dry mouth, hyperprolactinemia and abdominal pain. Olanzapine was associated to leukopenia and thrombocytopenia (FDA, 1996). Apart from metabolic effects, olanzapine causes short and long-term movement disorders, such as bradykinesia, akathisia; Parkinsonism and dystonia. These are known as extrapyramidal adverse drug reactions.

The most frequently reported adverse events during risperidone treatment are of greater prevalence (more than 10%) than metabolic effects, and are the following: insomnia, anxiety, headache, upper respiratory tract infection, Parkinsonism, depression and akathisia. The less frequent, but still common (1–10% of cases) adverse events are pneumonia, bronchitis, sinusitis, urinary tract infection, influenza; anemia, sleep disorders, agitation, decreased libido, sedation or sleepiness, dystonia, dizziness, dyskinesia, tremor, blurred vision, tachycardia, hypotension or hypertension, abdominal pain, abdominal discomfort, vomiting, nausea, constipation, gastroenteritis, diarrhea, dyspepsia, dry mouth, hyperprolactinemia, toothache, exanthema, muscle spasms, musculoskeletal pain, back pain, arthralgia (FDA, 1993).

The most common adverse drug reactions (>10% patients) to aripiprazole are mostly extrapyramidal effects, headache, agitation, insomnia, anxiety, nausea and vomiting, akathisia, light-headedness, and constipation (Prommer, 2017).

Dizziness seems to happen due to the antagonism on α1-adrenergic receptors, as well as tachycardia, bradycardia and syncope (FDA, 1996). Antagonism on muscarinic receptors causes anticholinergic effects, such as dry mouth and constipation (Bhana et al., 2001). Antagonism on 5-HT receptors seems also to be in involved in the appearance of constipation, as serotonin participates in the activation of colonic smooth muscle contraction (Zhang et al., 2016). Headache and somnolence are thought to be caused by the antagonism on 5-HT, H1 histamine and α1-adrenergic receptors, however, the molecular mechanisms involved are not completely known (Fang et al., 2016). Olanzapine seems to induce the least neurological adverse events compared to risperidone and aripiprazole (Cheng et al., 2019).

During antipsychotic treatment, the follow up of several adverse events is highly recommended, e.g. QTc interval monitoring, since some antipsychotics may be associated with QTc prolongation, such as thioridazine or ziprasidone, which might be a signal of increased risk of arrhythmia (Marder et al., 2004). Olanzapine, risperidone and aripiprazole do not usually alter the QTc length (Czekalla et al., 2001; Glassman and Bigger, 2001; Ozeki et al., 2010; Spellmann et al., 2018). Risperidone can cause hypotension (Wilson et al., 2017), while olanzapine and aripiprazole usually do not affect blood pressure (Woo et al., 2009; Seven et al., 2017). Some studies observed a lower systolic and diastolic blood pressure, heart rate and QTc lowering effect on the first day of olanzapine treatment, however these effects progressively diminished on the following days probably due to developing tolerance (Bever and Perry, 1998; Koller et al., 2021).

Antipsychotics, including olanzapine, risperidone and aripiprazole, are also known to cause neuroleptic malignant syndrome, a rare life threatening adverse drug reaction (FDA, 1993, 1996, 2014). The four main symptoms of this syndrome are altered mental status, muscle rigidity, hyperthermia, and autonomic instability (Velamoor, 2017). It is thought to be related with D2 receptor blockage, among others (Velamoor, 2017).

## Personalized Medicine

Personalized medicine is an emergent approach with the objective to prevent, diagnose and treat diseases using specific information about genes, proteins and environment of each patient individually (Di Sanzo et al., 2017). This field evolved rapidly during the last decade thanks to the continuous evolution of technology and to the development of genetic analyses (Di Sanzo et al., 2017).

Pharmacogenetics studies the influence of genetic variability on drug response. Its aim is to select "the right drug, at the right dose, for the right patient," predicting the tolerance and effectiveness of the drug in each patient, considering the individual's genetic information (Daudén Tello, 2006). To date, two of the most important groups that develop pharmacogenetic guidelines are the Clinical Pharmacogenetics Implementation Consortium (CPIC) (Caudle et al., 2014) and the Dutch Pharmacogenetics Working Group (DPWG) (Bank et al., 2018). The CPIC was established in 2009 as a joint project between the Pharmacogenomics Research Network (PGRN) and the Pharmacogenomics Knowledge Base (PharmGKB) (Caudle et al., 2014). These guidelines classify individuals into different metabolizer phenotypes for different enzymes based on the genetic variants in the gene of interest. The main phenotypes are PM, when individuals carry non function or decreased function variants; intermediate metabolizers (IM), when individuals carry decreased function variants or non-function variants with normal function variants, presenting higher activity than PMs; NM, when individuals carry normal function variants and ultrarapid metabolizers (UM), when individuals carry increased function variants. Moreover, the phenotype of specific patients can be modified if they are treated with inhibitors or inducers of the enzyme of interest. Pharmacogene Variation Consortium also gathers information about the polymorphisms identified in each pharmacogene (Pharmacogene Variation Consortium, 2017). Regulatory agencies such as FDA (Food and Drug Administration) and EMA (European Medicines Agency) already include pharmacogenetic dosing recommendations for different drugs (FDA, 2019; EMA, 2020).

Antipsychotic treatment shows a hardly replicable response that cannot be predicted, leading to a trial-and-error prescribing strategy to choose the ideal treatment for each patient (Lally and MacCabe, 2015). Genetic factors influencing pharmacokinetics and pharmacodynamics may be the cause of part of this heterogeneity (Eum et al., 2016). The DPWG published guidelines in favor of *CYP2D6* genotyping for patients under aripiprazole and risperidone treatment, whereas no recommendation has been published for olanzapine to date (Bank et al., 2018; PharmGKB guidelines, 2020). Regarding aripiprazole, the DPWG proposed that a reduced dose should be administered for CYP2D6 PMs (Swen et al., 2011). Additionally, an adjustment of the normal dose is recommended if the patient is treated with concomitant CYP3A4 inhibitors, inducers or CYP2D6 inhibitors (FDA, 2014). There are no dosage recommendations for olanzapine based on CYP2D6 phenotype. Since olanzapine is metabolized by CYP1A2, a lower starting dose should be considered in patients treated with fluvoxamine or other CYP1A2 inhibitors (FDA, 1996). Regarding risperidone, the DPWG recommends a reduction of risperidone dose in CYP2D6 PMs since May 2020. Additionally, it recommends changing treatment to an alternative drug or titration of the dose according to the maximum concentration for the active metabolite for CYP2D6 UMs (PharmGKB guidelines, 2020). Moreover, the proper dose for the patient has to be evaluated in case of coadministration of other drugs, such as CYP3A4 and ABCB1 inducers (carbamazepine, phenobarbital, etc.), CYP2D6 inhibitors (fluoxetine, paroxetine) or ABCB1 inhibitors (e. g. Verapamil) (FDA, 1993).

The findings of several studies show that pharmacogenetics may be useful in the clinical practice, mainly to reduce the adverse drug reactions to these drugs, and thus achieve greater adherence to treatment (Brandl et al., 2014; Zai et al., 2018).

## Pharmacogenetics of Olanzapine, Aripiprazole and Risperidone

Several approaches allow investigating if genetic variants are associated with pharmacokinetics and pharmacodynamics of antipsychotics. One approach is genome-wide association studies (GWAS), which can discover new genes without any prior hypothesis on the complex biology of the disease. The main drawbacks of GWAS are its high price and complexity. Moreover, careful statistical analysis must be performed in other to avoid false positives resulting from the massive amount of statistical tests. Additionally, the selection of the individuals included should be done carefully to avoid insufficient sample size and population stratification. GWAS are used to discover new genes involved in a disease or in a drug response. However, the findings made in GWAS have to be confirmed in candidate genes studies. Candidate gene studies are association studies with the objective to identify relationships between polymorphisms in candidate genes, also called pharmacogenes, and different clinical events, such as efficacy and safety in patients or pharmacokinetics and safety in healthy volunteers. Candidate genes are previously selected based on a suspected role or a previous association in the development of a specific disease or phenotype. The advantages of candidate gene studies are its simplicity and affordability. The main disadvantages are the considerable chance of not finding associations derived from the limited gene selection and no possible gene discovery. The majority of the data presented here was collected from candidate gene studies.

The discovery of pharmacogenetic biomarkers could improve antipsychotic treatment. The administration of the right drug at the right dose for each patient facilitates the achievement of the desired efficacy while lowering the risks of adverse effects, thus achieving a greater adherence to therapy and a better control of the disease.

### Genes Related to Pharmacokinetics

#### Olanzapine

Among the polymorphisms found in *CYP1A2*, only some of them were associated with variation in the expression or inducibility of the enzyme. Five alleles were defined that can affect olanzapine metabolism: *1C (rs2069514, −3860G > A), *1D (rs35694136, 22467delT), *1E (rs2069526), *1F (rs762551, −163C > A) and *1K (rs2069526, rs12720461, and rs762551). However, only the *1D and *1F alleles were associated with increased and reduced olanzapine plasma concentration, respectively, in some candidate gene studies (Söderberg and Dahl, 2013; Czerwensky et al., 2015), although this association could not be found in other candidate gene studies (Cabaleiro et al., 2013; Koller et al., 2020). A polygenic risk score model was proposed for *CYP1A2*, which classifies individuals into PMs, NMs and UMs (Saiz-Rodríguez et al., 2019). However, associations between this risk score model and pharmacokinetic parameters were not found in other candidate gene studies (Koller et al., 2020; Zubiaur et al., 2021) (Table 2). The rs2472297 polymorphism, located between the *CYP1A1* and *CYP1A2* genes, was associated with variations in olanzapine plasma concentration in a GWAS, explaining 2% of the variability (Söderberg and Dahl, 2013). The lack of agreement among studies means that there are still no recommendations for olanzapine dosing based on CYP1A2 genotype (Table 2).

**TABLE 2:** Genes associated with olanzapine pharmacokinetics or pharmacodynamics.

Olanzapine				
Pharmacokinetics				
Gene	Variant	Association	Associated	Not associated
CYP1A2	*1D	↑[Olanzapine]plasma	(Söderberg and Dahl, 2013; Czerwensky et al., 2015)	(Cabaleiro et al., 2013; Koller et al., 2020)
	*1F	↑[Olanzapine]plasma	(Söderberg and Dahl, 2013; Czerwensky et al., 2015)	(Cabaleiro et al., 2013; Koller et al., 2020)
CYP1A1-CYP1A2	rs2472297	↑[Olanzapine]plasma	Söderberg and Dahl, (2013)	
CYP2D6	UM	No effect		(Nozawa et al., 2008; Söderberg and Dahl, 2013; Koller et al., 2020
				Zubiaur et al. (2021)
	PM	↑Insulin levels	Koller et al. (2021)	
CYP3A5	*3/*3	↓AUC	Cabaleiro et al. (2013)	Zubiaur et al. (2021)
CYP2C9	PM	↑Adverse drug reactions	(Cabaleiro et al., 2013; Zubiaur et al., 2021)	
ABCB1	rs1045642	↑AUC	(Skogh et al., 2011; Saiz-Rodríguez et al., 2018)	
	rs2032582	↑AUC	(Skogh et al., 2011; Saiz-Rodríguez et al., 2018)	
	rs1128503	↑[Olanzapine]plasma	Skogh et al. (2011)	
	rs3842	↑Olanzapine exposure	Zubiaur et al. (2021)	
	Others	Variation T1/2	Koller et al. (2020)	
UGT1A4	*3	↑Glucuronidation	Erickson-Ridout et al. (2011)	
UGT2B10	*2	↓Glucuronidation	Erickson-Ridout et al. (2011)	
UGT1A1	rs887829	↑T_max_	Koller et al. (2020)	
FMO3	rs2266782-rs2266780	↓N-oxidation	Söderberg et al. (2013)	
FMO1	*6	↑[Olanzapine]plasma	Söderberg et al. (2013)	
**Pharmacodynamics**				
5-HTR2C	rs3813929	↓Weight gain	(Templeman et al., 2005; Sicard et al., 2010)	
		↓body Mass index		
	rs1414334	↑Risk of metabolic syndrome, ↑Weight gain	(Ma et al., 2014; Koller et al., 2021)	

	rs6318	↓Weight gain	(Sicard et al., 2010; Ma et al., 2014)	
	rs518147	↓Weight gain	Sicard et al. (2010)	
DRD2	rs1800497	↑Prolactin levels	(López-Rodríguez et al., 2011; Brennan, 2014; Zubiaur et al., 2021)	Koller et al. (2021)
		↑Adverse drug reactions		
	rs1799732	↑Weight gain	(Lencz et al., 2010; Zubiaur et al., 2021)	
		↑Adverse drug reactions		
DRD3	rs6280	↑Response to olanzapine, ↑Prolactin levels	(Brennan, 2014; Koller et al., 2021)	

APOC3	rs4520	↑Triglyceride levels	Koller et al., (2021)	

No-function and reduced-function were defined in CYP2D6 (Caudle et al., 2020). These alleles allow the classification of individuals into different phenotypes: PM, IM, NM and UM. Although this cytochrome is involved in the metabolism of olanzapine, numerous candidate gene studies did not find associations between phenotypes and plasma concentration of olanzapine (Nozawa et al., 2008; Söderberg and Dahl, 2013; Koller et al., 2020; Zubiaur et al., 2021). This is consistent with its secondary role on the metabolism of this drug (Table 2).

*CYP3A4* is known to be in involved in olanzapine metabolism (Söderberg and Dahl, 2013). *CYP3A4* presents high allelic variance (26 alleles), however, only a few of these alleles possess altered function (Pharmacogene Variation Consortium, 2017). Its most studied no-function alleles are CYP3A4*6 (rs4646438) and CYP3A4*20 (rs67666821). However, there is no clear evidence for their effects to date (Sanchez Spitman et al., 2017). Regardless the presence of no function variants, CYP3A4 could compensate for this effect by being overexpressed, as it is highly inducible (Sanchez Spitman et al., 2017). Its high inducibility makes it difficult to find pharmacogenetic associations with drugs (Sanchez Spitman et al., 2017). Due to the similar substrate specificity between CYP3A4 and CYP3A5, some drugs metabolized by CYP3A4 are also metabolized by CYP3A5. CYP3A5*3 (defined by rs776746) is a non-function allele (Wong, 2004). A study found significantly lower AUC in individuals *3/*3 for CYP3A5 (Cabaleiro et al., 2013). A different candidate gene study did not find differences in olanzapine pharmacokinetics regarding CYP3A4/5 (Zubiaur et al., 2021). Therefore, results are contradictory, and additional studies are needed to evaluate its impact on olanzapine metabolism (Table 2).

Additionally, two candidate gene studies found a higher T_1/2_ and Vd in CYP2C9 PM (*2/*3 and *3/*3 carriers) compared to IM and NM, and a higher incidence of adverse drug reactions in *3 carriers (Cabaleiro et al., 2013; Zubiaur et al., 2021) (Table 2).

Several polymorphisms were identified in the *ABCB1* gene; three of them were related to olanzapine pharmacokinetics in different candidate gene studies. 3435T > C (rs1045642) and 2677T > G/A (rs2032582) T allele carriers presented a higher plasma area under the curve (AUC) of olanzapine, as well as a better response in olanzapine treatment (Skogh et al., 2011; Saiz-Rodríguez et al., 2018). In addition, one study observed that 1236T > C (rs1128503) T allele carriers were associated with a higher serum concentration of olanzapine (Skogh et al., 2011). In a different candidate gene study, ABCB1 rs3842 CC individuals showed a greater olanzapine exposure (Zubiaur et al., 2021). Other polymorphisms were related to a variation of olanzapine T_1/2_ (Koller et al., 2020) (Table 2).

Two alleles were identified in the *UGT1A4* gene: *2 (*p*.P24T, rs6755571) and *3 (*p*.L48V, rs2011425). The *3 allele was associated with increased glucuronidation of olanzapine *in vivo*. *UGT2B10* *2 (*p*.D67Y, rs61750900) was associated with a decreased olanzapine glucuronidation *in vitro* (Erickson-Ridout et al., 2011). A candidate gene study found that UGT1A1*80 (rs887829) *80 homozygotes had higher T_max_ than *1/*80 and *1/*1 individuals (Koller et al., 2020) (Table 2).

The three most studied *FM O 3* polymorphisms are *p*. E158K (rs2266782), *p*. V257M (rs1736557) and *p*. E308G (rs2266780). *p*. E158K and *p*. E308G constitute the allele K158-G308 (Söderberg and Dahl, 2013). This allele was associated with a reduction of olanzapine N-oxidation *in vitro*. The *6 allele (rs12720462) in *FM O 1* gene seems to be associated with higher olanzapine plasma concentrations (Söderberg et al., 2013) (Table 2).

#### Aripiprazole

The only existing pharmacogenetic guideline in aripiprazole treatment is for *CYP2D6*. The DPWG and the Food and Drug Administration (FDA) recommend dose reduction for CYP2D6 PM (Swen et al., 2011; FDA, 2014, 2016). This group presents higher C_max_ and T_1/2_ (Kinghorn and McEvoy, 2005; FDA, 2014; Belmonte et al., 2018) (Table 3).

**TABLE 3:** Genes associated with aripiprazole pharmacokinetics or pharmacodynamics.

Aripiprazole				
Pharmacokinetics				
Gene	Variant	Association	Associated	Not associated
CYP2D6	PM	↑Cmax, ↑T1/2	(Kinghorn and McEvoy, 2005; Belmonte et al., 2018)	
CYP3A4	*6	No effect		Sanchez Spitman et al. (2017)
	*20	No effect		Sanchez Spitman et al. (2017)
	*26	No effect		Sanchez Spitman et al. (2017)
CYP3A5	*3/*3	↑[Aripiprazole]plasma	Suzuki et al. (2014)	Belmonte et al. (2018)
ABCB1	rs1045642	↓[Aripiprazole]plasma	Rafaniello et al. (2018)	(Suzuki et al., 2014; Belmonte et al., 2018; Koller et al., 2018)
	rs2032582	↓[Aripiprazole]plasma	(Belmonte et al., 2018; Rafaniello et al., 2018)	(Suzuki et al., 2014; Belmonte et al., 2018; Koller et al., 2018)
		↓Cl		
	rs1128503	↓Cl	Belmonte et al. (2018)	
**Pharmacodynamics**				
5-HTR2A	rs6311	↓Response for negative symptoms	Chen et al. (2009)	
DRD2	rs1800497	↑Response for negative symptoms	(Kwon et al., 2008; Ramsay et al., 2015)	
		↓Cognitive performance		
	rs6277	↓Response to aripiprazole, ↓Cognitive performance	(Shen et al., 2009; Ramsay et al., 2015)	

CYP3A4 is aripiprazole’s secondary metabolizing enzyme. Patients should receive a reduced dose when concomitant CYP3A4 inhibitors are prescribed (FDA, 2014). Since it is highly inducible (Sanchez Spitman et al., 2017), it is difficult to find associations between polymorphisms and drug pharmacodynamics or pharmacokinetics.

Due to similarity between CYP3A4 and CYP3A5 families (Huang et al., 2004), other genes could be involved in aripiprazole metabolism, e.g., CYP3A5. CYP3A5 *3/*3 carriers do not express CYP3A5, therefore, higher aripiprazole concentrations could be expected (Lamba et al., 2012). However, results are contradictory. Some candidate gene studies reveal no effect (Suzuki et al., 2014), while other reports its involvement in the development of adverse drug reactions (Belmonte et al., 2018) (Table 3).

Other gene studied in aripiprazole pharmacokinetics is *ABCB1* (Kirschbaum et al., 2010; Saiz-Rodríguez et al., 2018; Koller et al., 2020). Among the different mutations of *ABCB1*, carriers of the two mutations 2677T > G/A (rs2032582) and 3435T > C (rs1045642) presented lower plasma concentration (Rafaniello et al., 2018). Nonetheless, these results are controversial since other candidate gene studies reported no differences between *ABCB1* variants (Suzuki et al., 2014; Belmonte et al., 2018; Koller et al., 2018). In another study, carriers of the rs2032582 and rs1128503 polymorphisms affected aripiprazole Cl (Belmonte et al., 2018) (Table 3).

#### Risperidone

PMs for CYP2D6 showed a higher risperidone C_max_, AUC and T_1/2_ and a lower Cl (Jovanović et al., 2010; Cabaleiro et al., 2014) and a lower 9-hydroxyrisperidone AUC, C_max_ and a higher T_max_ (Cabaleiro et al., 2014). A different candidate gene study observed a higher plasma concentration risperidone/9-hydroxyrisperidone ratio in IM compared to NM and UM (Vanwong et al., 2016). Similar results were found in healthy volunteers (Novalbos et al., 2010). According to these results, children who are PM or IM for CYP2D6 have higher risk of suffering from adverse effects when treated with risperidone (Oshikoya et al., 2019) and extrapyramidal effects are more probable in IM patients (Ito et al., 2018). However, the active moiety levels are not always affected by CYP2D6 phenotype (Kang et al., 2009) (Table 4).

**TABLE 4:** Genes associated with risperidone pharmacokinetics or pharmacodynamics.

Risperidone				
Pharmacokinetics				
Gene	Variant	Association	Associated	Not associated
CYP2D6	IM	↑[Risperidone]plasma	(Novalbos et al., 2010; Ito et al., 2018; Oshikoya et al., 2019)	Kang et al. (2009)
		↑Risperidone/9-hydroxyrisperidone ratio		
		↑Adverse effects, ↑Extrapyramidal effects		
	PM	↑Cmax, ↑Tmax	(Jovanović et al., 2010; Cabaleiro et al., 2014; Oshikoya et al., 2019)	Kang et al. (2009)
		↑Adverse effects		
		↓9-hydroxyrisperidone AUC		
CYP3A5	*3/*3	↑[Risperidone]plasma, ↑[9-hydroxyrisperidone]plasma, ↑Active moiety	Kang et al. (2009)	(Vandenberghe et al., 2015; Ganoci et al., 2016)
ABCB1	rs1045642	↑Weight gain, ↑Extrapyramidal symptoms, ↑Risperidone and 9-hydroxyrisperidone Cl	(Kuzman et al., 2008; Jovanović et al., 2010; Saiz-Rodríguez et al., 2018)	(Xing et al., 2006; Yasui-Furukori et al., 2007)
	rs2032582	↑Weight gain	(Kuzman et al., 2008; Jovanović et al., 2010)	(Xing et al., 2006; Yasui-Furukori et al., 2007)
		↑Extrapyramidal symptoms		
	rs1128503	↑Response to risperidone	Xing et al. (2006)	
COMT	rs9606186	↑Efficacyy in male patients	Zhao et al. (2012)	
	rs165599	↑Efficacy on negative symptoms	Han et al. (2017)	
**Pharmacodynamics**				
5-HTR2C	rs3813929	No effect		(Ikeda et al., 2008; Kuzman et al., 2008; Alladi et al., 2019)
	rs518147	No effect		Ikeda et al. (2008)
5-HTR2A	rs6313	↑Response for negative symptoms	Lane et al. (2002)	Alladi et al. (2019)
	rs6311	No effect		Alladi et al. (2019)
DRD2	rs1800497	↑Improvement in PANSS scale, ↑Prolactin levels	(López-Rodríguez et al., 2011; Brennan, 2014)	Alladi et al. (2019)
	rs1799732	No effect		Alladi et al. (2019)
	rs1801028	↑Efficacy on negative symptoms	Han et al. (2017)	
	rs1799978	↑Improvement in PANSS scale	Ikeda et al. (2008)	
	rs1801028	↑Response to risperidone	Lane et al. (2004)	
DRD3	rs6280	↑Response to risperidone	Firouzabadi et al. (2017)	Ikeda et al. (2008)

CYP3A4 and CYP3A5 are also involved in risperidone metabolism. CYP3A5 non-expressors exhibited higher plasma concentrations of both risperidone and 9-hydroxyrisperidone and a higher active moiety than its expressors (Kang et al., 2009). However, two different candidate gene studies did not find differences in concentrations of risperidone, 9-hydroxyrisperidone and the active moiety regarding the CYP3A phenotype (Vandenberghe et al., 2015; Ganoci et al., 2016). Further studies are needed to estimate the influence of CYP3A phenotype on risperidone pharmacokinetics (Table 4).

A candidate gene study showed that schizophrenic women who were G for rs2032582 (2677T > G/A) and C for rs1045642 (3435T > C) for *ABCB1* were less represented in the group of patients who suffered a greatest weight gain in risperidone treatment (Kuzman et al., 2008). In a different study, rs1045642T carriers and T carriers in rs2032582 and rs1045642 suffered from extrapyramidal symptoms with a higher frequency (Jovanović et al., 2010). For this reason, it was proposed that rs1045642T and rs2032582T carriers reach a higher risperidone concentration in the brain, allowing stronger effects of this antipsychotic, including its adverse effects. T carriers in the rs1045642 single nucleotide polymorphism (SNP) were found to have a higher risperidone and 9-hydroxyrisperidone Cl (Saiz-Rodríguez et al., 2018). A different candidate gene study did not find differences in risperidone and 9-hydroxyrisperidone plasma concentrations depending on rs2032582 and rs1045642 *ABCB1* polymorphisms (Yasui-Furukori et al., 2007), and another one did not find differences in risperidone response (Xing et al., 2006). However, this last study found a better response to risperidone in T/T rs1128503 (1236T > C) patients (Xing et al., 2006). Different candidate gene studies obtained different results regarding these polymorphisms and risperidone pharmacokinetics. For this reason, further studies should be conducted to clarify the impact of these SNPs in risperidone pharmacokinetics (Saiz-Rodríguez et al., 2018) (Table 4).

The catechol-O-methyl transferase (COMT) enzyme is involved in the regulation of dopamine availability in the brain. Some studies found that some *COMT* polymorphisms influenced antipsychotics response. In the treatment with risperidone, rs9606186 was associated with clinical efficacy in male patients (Zhao et al., 2012), while rs165599 was significantly associated with risperidone efficacy on negative symptoms (Han et al., 2017) (Table 4).

### Genes Related to Pharmacodynamics

#### Olanzapine

Several studies showed that the *HTR2C* −759C > T (rs3813929) T allele has a protective effect against olanzapine-produced weight gain (Templeman et al., 2005). The C allele of the rs1414334 intronic polymorphism is associated with an increased risk for developing metabolic syndrome and a higher weight gain after olanzapine treatment (Ma et al., 2014; Koller et al., 2021). This SNP is in complete linkage disequilibrium with the rs6318 (*p*.Cys23Ser) polymorphism, where the Ser allele is constitutively more active (Ma et al., 2014). Carriers of T, C and Ser alleles for the −759C > T, −697G > C (rs518147) and Cys23Ser, presented lower weight gain and body mass index (BMI) (Sicard et al., 2010) compared to the other alleles (Table 2).

Genetic variations in the *DRD2* gene caused different responses to olanzapine treatment (Houston et al., 2011). The TaqIA (rs1800497) T allele seems to be associated with increased prolactin levels during olanzapine treatment (López-Rodríguez et al., 2011; Brennan, 2014). The InDel polymorphism rs1799732 is located in the gene promoter. Individuals who presented the deletion suffered greater weight gain when treated with olanzapine than those without the deletion (Lencz et al., 2010). This variation in the *DRD2* promoter region may produce differences in the sensitivity of the receptor to the effects of antipsychotics on reward signals associated with food intake and satiety (Lencz et al., 2010). A different candidate gene study also found a higher incidence of adverse reactions in mutant individuals for TaqIA and rs1799732 (Zubiaur et al., 2021) (Table 2).

The presence of Gly in *DRD3* rs6280 (*p*.Ser9Gly) is associated with a better response to olanzapine (Brennan, 2014). Moreover, the Gly allele was related to a higher increase in prolactin levels compared to Ser allele (Koller et al., 2021) (Table 2).

The apolipoprotein C3 (*APOC3*) rs4520 C/C genotype was associated with higher triglyceride concentrations after olanzapine administration in a candidate gene study (Koller et al., 2021). Polymorphisms in this gene influence plasma triglyceride levels since the APOC3 protein increases plasma triglyceride levels by the inhibition of lipoprotein lipase, stimulates low-density lipoprotein secretion and intestinal triglyceride trafficking modulation (Koller et al., 2021). A different study observed lower T_1/2_ in G/G rs5128 *APOC3* (Zubiaur et al., 2021) (Table 2).

#### Aripiprazole

There is no extensive evidence on the influence of polymorphisms in aripiprazole pharmacodynamics. One of the most studied variants in antipsychotic pharmacodynamics is the *DRD2* TaqIA polymorphism. The *A1 allele corresponds with lower density of DRD2 receptors in the striatum (Jönsson et al., 1999). Some groups reported better aripiprazole response for *A1 allele carriers (Kwon et al., 2008). Furthermore, C/C homozygotes for rs6277 in *DRD2* gene present poorer response to aripiprazole (Shen et al., 2009). Also, carriers of C allele in rs6277 and *A1 allele in Taq1A had poorer cognitive performance (Ramsay et al., 2015) (Table 3).

Other reported genetic variants are mutations of serotonin receptor such as 5-*HTR*_*2A*_. The different variants of this receptor present different binding affinities for aripiprazole (Davies et al., 2011). Concretely, subjects with the G/G C/C genotype of *HTR2A* rs6311 (1438G > A/T) and rs6313 (102T > C) polymorphisms showed poorer aripiprazole response for negative symptoms in a candidate gene study (Chen et al., 2009) (Table 3).

#### Risperidone

*DRD2* rs1801028 (*p*.Ser311Cys) was significantly associated with risperidone efficacy on the treatment of negative symptoms (Han et al., 2017). In addition, being heterozygous for the rs1801028 variant in *DRD2* was associated with a better response to risperidone compared to Ser homozygous (Lane et al., 2004). Taq1A and rs1799732 genetic variants were not associated with antipsychotic to risperidone in one candidate gene study (Alladi et al., 2019). However, a different candidate gene study found an association between *A1/*A1 genotype for TaqIA and A/A genotype for rs1799978 SNP and a higher improvement in PANSS scale (Ikeda et al., 2008). The TaqIA*A1 allele seemed to be associated with increased prolactin levels during risperidone treatment (López-Rodríguez et al., 2011; Brennan, 2014) (Table 4).

There are controversial results regarding the influence of the rs6280 polymorphism on *DRD3* on risperidone response. A candidate gene study with autistic children observed a better risperidone response in Gly allele carriers (Firouzabadi et al., 2017). However, a different candidate gene study with schizophrenic patients did not find this association (Ikeda et al., 2008) (Table 4).

Another candidate gene study observed that *HTR2A* rs6313 C/C patients showed a superior risperidone response particularly for negative symptoms (Lane et al., 2002). However, a different candidate gene study did not find associations between *HTR2A* rs6311 and rs6313 polymorphisms and risperidone response (Alladi et al., 2019). In a candidate gene study carried in schizophrenic women, *HTR2C* rs3813929 (−759C > T) polymorphism was not found to be associated to a higher weight gain (Kuzman et al., 2008) or to a better response (Ikeda et al., 2008; Alladi et al., 2019). Neither was the −697C > G SNP associated with a better risperidone response (Ikeda et al., 2008) (Table 4).

## Conclusion

Based on our review, olanzapine induces more metabolic adverse drug reactions than risperidone and aripiprazole, being aripiprazole the drug that induces the least metabolic adverse reactions. The goal of personalized medicine is to predict the side effects of each drug in each patient individually. In order to reach this goal, specific biomarkers, i.e. genetic polymorphisms should be tested before starting the treatment. Only a few polymorphisms were found to be associated to olanzapine, aripiprazole and risperidone treatment outcomes. Since olanzapine is metabolized by CYP1A2, a lower starting dose should be considered in patients treated with fluvoxamine or other CYP1A2 inhibitors. Regarding aripiprazole, a reduced dose should be administered for CYP2D6 PMs. Additionally, a reduction to a quarter of the normal dose is recommended if the patient is treated with concomitant CYP3A4 inhibitors. For risperidone, a dose adjustment should be evaluated in case of coadministration of CYP3A, CYP2D6 and ABCB1 inhibitors. Although several studies found possible associations with the metabolic adverse drug reactions of these drugs, to date, no genetic polymorphism is recommended for testing. Consequently, this deficit should be addressed to offer better treatment to patients under antipsychotic therapy. This review lists all of the possible targets which have been found to date.
